# The Canadian Cardiac Rehabilitation Registry: Inaugural Report on the Status of Cardiac Rehabilitation in Canada

**DOI:** 10.1155/2015/278979

**Published:** 2015-08-18

**Authors:** Sherry L. Grace, Trisha L. Parsons, Kristal Heise, Simon L. Bacon

**Affiliations:** ^1^School of Kinesiology and Health Science, Faculty of Health, York University, Bethune 368, 4700 Keele Street, Toronto, ON, Canada M3J 1P3; ^2^GoodLife Fitness Cardiovascular Rehabilitation Unit, University Health Network, Toronto Western Hospital, 8e-402, Toronto, ON, Canada M5T 2S8; ^3^School of Rehabilitation Therapy, Faculty of Health Sciences, Queen's University, 31 George Street, Louise D. Acton Building, Room 208 CEC, Kingston, ON, Canada K7L 3N6; ^4^Canadian Association of Cardiovascular Prevention and Rehabilitation, 1390 Taylor Avenue, Winnipeg, MB, Canada R3M 3V8; ^5^Department of Exercise Science, Concordia University, 7141 Sherbrooke Street West, SP 165-35, Montreal, QC, Canada H4B 1R6; ^6^J-135 Centre de Réadaptation Jean-Jacques Gauthier, Hopital du Sacre-Coeur de Montreal, 5400 Boulevard Gouin Ouest, Montreal, QC, Canada H4J 1C5

## Abstract

*Introduction*. There are over 200 Cardiovascular Rehabilitation (CR) programs in Canada, providing services to more than 50,000 new patients annually. The objective of this study was to describe the impact of CR in Canada.* Methods*. A retrospective analysis of Canadian CR Registry data is presented. There were 12 programs participating, with 4546 CR participants.* Results*. The average wait time between patient referral and CR admission was 68 ± 64 days. Participants were 66.3 ± 11.5 years old, 71% male, and 82% White. The three leading referral events were coronary artery bypass graft surgery, percutaneous coronary intervention, and acute coronary syndrome. At discharge, data were available for ~90% of participants. Significant improvements in blood pressure (systolic pre-CR 123.5 ± 17.0, post-CR 121.5 ± 15.8 mmHg; *p* < .001), lipids, adiposity, and exercise capacity (peak METs pre-CR 6.5 ± 2.8, post-CR 7.2 ± 3.1; *p* < .001) were observed. However, target attainment for some risk factors was suboptimal.* Conclusions*. This report provides the first snapshot of the beneficial effects of CR in Canada. Not all patients are equally represented in these programs, however, leaving room for more referral of diverse patients. Greater attainment of risk reduction targets should be pursued.

## 1. Introduction

Cardiovascular disease (CVD) is a leading cause of death and disability globally [1]. Effective prevention and management of CVD requires multifactorial behavioural and risk factor management. Cardiovascular Rehabilitation (CR) is a comprehensive outpatient chronic disease management program designed to improve CV health [2]. CR programs offer medical assessment, structured programs of exercise training, patient and family education, and the delivery of CV risk factor management strategies. Participation in CR reduces all-cause mortality by approximately 15% and CV mortality by 25% when compared to usual care [3].

There are over 150 CR programs in Canada, providing services to more than 50,000 new patients annually [4]. The Canadian Association of Cardiovascular Prevention and Rehabilitation has guidelines on the appropriate structure and expected outcomes of these programs [2]. However, there is a dearth of data on the exact nature of the CR services delivered in Canada and how effective these are at improving patient health. Consistent with other areas in medicine [5–7], the creation of an appropriately constructed, nationally representative registry can be a key mechanism for tracking the effectiveness of CR.

*The Canadian Cardiac Rehab Registry*. In response to the outcomes of the Ontario CR Pilot Project, [8] and subsequent recommendations from the Canadian Heart Health Strategy and Action Plan [9], vision for the Canadian Cardiac Rehab Registry (CCRR) was established in 2005. The goals are (1) to enable CR programs to benchmark performance, (2) to facilitate guideline adherence and, in turn, improve patient outcomes, (3) to build a clinical research database to study CR programming in Canada, and (4) to influence health policy.

The CCRR Committee, a Subcommittee of the Canadian Association of Cardiovascular Prevention and Rehabilitation's Board of Directors, was formed to create and operationalize a plan for the build and roll-out of the CCRR. Since its establishment, the CCRR Committee has continued to provide leadership and direction while overseeing Subcommittee work in the areas of Data Transfer Compatibility Verification, Program Liaison, and Research. Specifically, the Research Subcommittee has developed data access, data quality, and publications and presentations policies for the CCRR [10].

The goal of this inaugural CCRR report is to provide a summary of the impact of CR on the health of Canadians with CVD. In addition, aggregate data on the reach and the process of CR delivery within Canada will be presented.

## 2. Methods

The study was retrospective and observational in design. Each program offers CR in accordance with Canadian CR guidelines [2]; however there is some variation in terms of the professions represented on staff and the frequency and duration of the exercise sessions.

### 2.1. Measures

The CCRR tracks data collected in CR programs across Canada using an online database (https://registry.cacr.ca/). Programs are to provide data on all patients consecutively. Capturing data at CR program intake and discharge, approximately 200 data elements are collected on each patient. This includes (1) patient sociodemographic and clinical characteristics, (2) key dates such as those of the index event, referral, intake, and discharge, (3) risk factor status at intake assessment, (4) interim events and program utilization, (5) clinical data from intake and discharge assessments such as stress test results, risk factors, and psychosocial well-being, and (6) medications. A standardized data dictionary is provided for each program (see http://www.cacpr.ca/resources/registry.cfm; see Supplemental Appendix in Supplementary Material available online at http://dx.doi.org/10.1155/2015/278979). For example, as shown, exercise stress tests are to be symptom-limited, maximal tests using a standardized protocol such as a modified Bruce [11] (pages 16–20), and time windows for intake and discharge assessments are provided (i.e., within 1 month). Information on lipid assessment is shown on pages 13-14 [12]. Programs provided the option of several psychosocial questionnaires to administer to patients, namely, the Hospital Anxiety and Depression Scale [13], the Beck Depression Inventory [14], and the Medical Outcomes Study SF-12 or SF-36 (see pages 23–25) [15].

The CCRR data abstraction tool includes predefined logic features and user alerts to identify potentially invalid data formats or values. Some variables are mandatory for the patient's file to be regarded as complete, and for the patients' data to uploaded into the registry. Edit checks are used to identify inconsistent or out-of-range data and prompt the user to correct or review data entries that are outside a predefined range.

Each participant in CR program nominates a data steward, whose responsibilities include the uploading or direct entry of data to the CCRR web-based interface. All stewards using the CCRR data abstraction tool receive individual passwords to create an audit trail for data entered or changed. Training in the use of the tool is provided for all users (e.g., web session to review the interface and data dictionary). CR program data stewards are able to enter data concurrently during the program or may enter data after patient discharge.

With regard to data quality, the data steward is responsible for working with CCRR staff and verifying that the data has been accurately collected and entered into the CCRR. The CCRR database analyst undertakes quarterly data audits, exploring missing data, and multivariate outliers, for example. These are then reviewed by the Research Subcommittee, for action. In order to monitor the quality of data entered into the CCRR, the programs must agree that CCRR staff may from time to time conduct on-site audits of data and collection procedures. Corrective action of identified errors must be rectified within the CCRR within 3 months of the audit.

### 2.2. Statistical Analysis

The current study includes all data collected from the inception of the national Registry in 2011 until February, 2013. Key data elements from the CCRR are described using standard descriptive analyses.

## 3. Results

### 3.1. Program Characteristics

Of the known 154 CR programs in Canada, there are 12 (7.8%) participating in the CCRR. Of these, 6 (50.0%) are from Ontario, 4 (33.3%) are from New Brunswick, and one (8.3%) each are from Nova Scotia and British Columbia. Of these, 25% would be considered academic programs. There are 4546 participants entered in the CCRR, with a mean of 603.6 ± 1059.6 (standard deviation) participants per site (range 38–3525; median = 190.5).

The mean wait time between patient referral and patient admission to CR program was 68 ± 64 (standard deviation) days (range = 0–986; median = 54). The majority of the participants (86%) travelled 30 minutes or less to attend CR. An additional 10% of participants travelled between 31–45 minutes. Only 3% of participants travelled more than 1 hour to CR.

### 3.2. Patient Characteristics

At the time of program admission, CCRR participants were on average 66.3 ± 11.5 years, and 71% were male. Most participants (62%) had a highest educational attainment of high school. Almost three-quarters (71%) identified their marital status as “married,” with most participants living with their spouse. Ethnicity is reported in Table 1.

The three leading CR referral events were coronary artery bypass graft surgery (15%), percutaneous coronary intervention (10% bare metal and 9% drug-eluting stent), and acute coronary syndrome (10% ST-elevation myocardial infarction and 10% non-ST elevation myocardial infarction). Other referral indications included heart failure, valve surgery, rhythm devices or posttransplant, stroke, or transient ischemic attack, peripheral vascular disease, and high-risk primary prevention patients. The risk factor status of CCRR participants at program intake is shown in Table 2.

### 3.3. Patient Outcomes: The Effects of CR

The mean number of days between CR intake and discharge was 177.4 ± 109.3 (median = 180). CR discharge data was available for approximately 90% of participants. The reason for premature termination was provided for 4% of patients. This was most often due to patient dropout (87%), followed by a noncardiac clinical event (9%), a cardiac event (3%), or death (1%).

Approximately 1% of patients experienced a major adverse cardiac event (excluding death) during CR. This was most often acute coronary syndrome (28%), percutaneous coronary intervention (22%), heart failure (21%), and bypass surgery (14%).

The comparison between CR intake and discharge measurements among participants with data at both assessment points is displayed in Table 3. The protocol used for the majority of the exercise tests was the Bruce (78%), followed by a modified Bruce (11%) [11]. The assessment of depressive and anxiety symptoms was most often undertaken through administration of the Hospital Anxiety and Depression Scale (HADS) [13]. As shown, there were statistically significant reductions in risk factor burden (i.e., blood pressure, lipids, and adiposity), as well as depressive symptoms, and improvements in exercise capacity.

At CR discharge, 15% of patients were at the guideline-recommended LDL-C target, 22% at target HDL, 81% at target triglycerides, and 96% were at target for total cholesterol recommendations [12]. With regard to blood pressure, 66% were less than 130/80 mmHg at discharge [17]. Moreover, 18% had a body mass index below 25 [16]. Finally, 11% of men and 22% of women had a waist circumference below recommended targets [18].

## 4. Discussion

This report provides the first snapshot of the pragmatic effect of CR in Canada. This may help to advance the care of Canadians with CVD. Overall results suggest that revascularization and acute coronary syndrome patients are accessing CR approximately 2 months after event/procedure, that they participate in programs that are within a 30-minute “acceptable” [19, 20] drive-time from their homes, and that participation results in significant improvements in risk factors and exercise capacity.

Of interest, while the median wait times from program referral receipt to program admission were within the “acceptable” benchmark established in Canada of 60 days [19], the benchmark considers the number of days from referral* event*, which was not captured herein. Indeed, in working towards national CR quality indicators [21], the CR community has explicitly operationalized key indicators such as wait times, including specification of exclusions, and will work to ensure these definitions are congruent and embedded in the CCRR. However, recent research suggests that CR initiation earlier than what was observed herein is safe and results in greater program enrolment [22]. Ultimately CR programs may need to adopt strategies to reduce wait times and better identify and address reasons for delay.

This snapshot also suggests that White, married males continue to be overrepresented in CR programs. Given that more universal referral and endorsement can mitigate these inequities [23], this is an area that the physician community can work to improve. In addition to reaching out to referral sources to ensure diverse patients are referred, the CR community needs to ensure that diverse patients feel welcome in our programs and that our offerings reflect their needs [24].

As expected, CR patients had a high burden of risk factors, though these were significantly reduced through program participation. The pre-CR values for blood pressure were already quite close to targets [17, 25]; however significant improvements were nevertheless achieved (although the clinical significance of these improvements is likely modest at best). The indicators for adiposity revealed pervasive and persistent obesity. Indeed, it has previously been suggested that CR programs may not be effective in tackling obesity [26]. There are some trials in the literature, however, of targeted weight reduction strategies in CR, which demonstrate that clinically-significant reductions can be achieved [27]. On the other hand, over half a metabolic equivalent of task (MET) increase was achieved, which has been shown to be clinically meaningful [28, 29]. Clearly this is an area where CR program communication with primary care could facilitate long-term approaches to reduce patient adiposity post-CR.

The LDL values at exit were approximately 2.0 mmol/L, while current Canadian guidelines recommend a target of ≤ 2.0 [12]. While this suggests the target has not been met, the current Canadian guidelines recommend that, in the presence of more severe baseline dyslipidemia or in patients whom therapy is limited by drug intolerance (statins were contraindicated in 0.5% of patients), a 50% or greater reduction of LDL-C from baseline is recommended, or to consider apoB. A 13% reduction was achieved overall. Unfortunately, the CCRR does not capture “baseline” lipid values, as likely the referral event was not the initial diagnostic point when statin therapy was initiated for the patient, or apoB. A previous Cochrane review identified randomized controlled trials comparing comprehensive CR to usual care on the outcome of LDL [30]. There was a significant net reduction of 0.51 mmol/L (95% CI = −0.82–0.19) with CR.

There is another established CR registry in the United Kingdom called the National Audit of Cardiac Rehabilitation (NACR; http://www.cardiacrehabilitation.org.uk/nacr/index.htm). These findings are fairly consistent with theirs. For instance, the average age of the patients referred to CR in NACR was 65 for men and 70 for women. Of those referred, 70% were male, 70% were married, and 82% were “White.” The primary referral indications were percutaneous coronary intervention, and acute coronary syndrome or myocardial infarction, followed by bypass graft surgery. Over one-fifth (22%) had comorbid diabetes, and 12% were smoking. The median wait in days to initiate CR was 57. Half of the patients achieved an LDL-C ≤ 2 by program exit, and significant reductions in waist circumference were observed. On the other hand, the burden of depressive and anxious symptoms was much higher in the UK (17% reporting elevated depression and 30% elevated anxiety).

Indeed, the NACR has the longest history of any CR registry internationally. Several nations in Europe also have registries [31], and there has been much interest recently in integrating these into the European Cardiac Rehabilitation Database (EuroCaReD; http://www.escardio.org/communities/EACPR/news/Pages/european-cardiac-rehabilitation-database.aspx). The American Association of Cardiovascular Prevention and Rehabilitation has most recently initiated a registry as well (https://www.aacvpr.org/Resources/OutpatientDataRegistries/OutpatientCardiacRehabDataRegistry/tabid/422/Default.aspx), since the CCRR was rolled out on a nation-wide scale in 2010. The leadership of these registries has been engaging in informal communications quarterly for the past year to share best practices.

Unfortunately the adoption of the CCRR by programs in Canada has been lower than desired. Members of the CCRR committees continue to invite programs to share their data with the CCRR. Webinars on the CCRR are offered regularly to interested programs. While there is currently no cost to join, program staff cite barriers to joining the registry. These include chiefly administrative hurdles in terms of privacy and agreement signatures and lack of human resources for data entry. Where programs have electronic databases, at a small cost, programs can match their variables to CCRR variables and then software programmers can “push” the data to the CCRR at quarterly intervals, hence mitigating the need for manual data entry. NACR has achieved much higher adoption, as the registry is recognized as means to demonstrate program quality to payers. Such a model should be explored in Canada.

Caution is warranted when interpreting this data, however, primarily due to generalizability limitations and missing data. With regard to the former, these findings are limited to the 4 of 10 provinces where CR programs are contributing data. With regard to the CCRR uptake within provinces, the penetration in New Brunswick is very high (~31% of programs participating) but is lower in Nova Scotia (~20%) and Ontario (~12%). While there are national CR guidelines, reimbursement of CR care varies widely by province and it would be expected that there would be some provincial variation in programs, as is observed for acute cardiac care [32]. As outlined above, the results are also limited in generalizability as the proportion of participating programs is low, and their representativeness in comparison to CR programs nationally is unknown.

Second, the CCRR Research Subcommittee has noted a lack of completeness of data submission as an issue threatening the quality of the data. Therefore, the sample sizes for the patient outcomes reported in Table 3 vary considerably. To this end, we have developed and enacted a data quality policy so that issues related to the CCRR interface as well as human factors can be addressed to ensure the rigor of CCRR data. The research committee is also exploring implementation of a minimum data set, to promote greater data completeness for key metrics. Finally, the technique to assess waist circumference was not explicitly stated in the data dictionary, and hence some variability in values may be due to measurement error.

In conclusion, this first snapshot of CR in Canada supports the beneficial effects of these programs. We encourage the research community to request access to this comprehensive registry, so that the data can be fully exploited and we will achieve a more comprehensive understanding of CR in Canada. Given that other countries have also initiated CR registries, in the future it may be possible not only to understand and improve patient outcomes in the Canadian context, but also to share best practices internationally.

## Supplementary Material

The Supplementary Material consists of the data dictionary 1.0 for the Canadian Cardiac Rehabilitation Registry. This is a listing of all variables assessed in the registry, with their definition (i.e., data sources, time windows, units of measurement). There are variables which are captured at cardiac rehabilitation intake assessment only, as well as variables which are assessed at both the intake and discharge assessments.

## Figures and Tables

**Table 1:** Ethnicity of CCRR participants.

	%	*N*
Aboriginal	1%	45
Arab/West Asian	7%	364
Black	5%	273
Chinese	4%	227
Korean	1%	45
Latin American	6%	318
South Asian	1%	55
Southeast Asian	5%	273
White/Caucasian	70%	3728

**Table 2:** Patient risk factor status at program intake.

Risk factor	*n* (%)
Abdominal obesity^*∗*^	3853 (89.0%) men
	3377 (78.0%) women
Overweight or obese^†^	3550 (82.0%)
Hyperlipidemia	2819 (81.0%)
Hypertension	2682 (61.8%)
Family history of CAD	2273 (52.4%)
Sedentary lifestyle	1682 (38.8%)
Diabetes^§^	1000 (23.1%)
Current smokers	771 (17.8%)

*Note*. CAD: coronary artery disease.

^*∗*^Waist circumference measured in centimeters. Thresholds as per American Heart Association: women >88 cm, men >102 cm [16].

^†^Body mass index above 25 kg/m^2^ [17].

^§^Medical chart documented diagnosis, or on therapy for diabetes.

**Table 3:** Intake to discharge comparisons in outcome measures among patients completing CR.

	CR intake	CR discharge	*p* ^†^
Blood pressure (mmHg)			
Systolic	123.51 ± 16.96	121.51 ± 15.79	*∗∗∗*
Diastolic	72.49 ± 10.50	72.09 ± 10.17	*∗∗∗*
Lipids (mmol/L)			
Low-density lipoprotein	2.32 ± 1.00	2.03 ± 0.88	*∗∗∗*
High-density lipoprotein	1.15 ± 0.35	1.17 ± 0.37	*∗∗∗*
Triglycerides	1.77 ± 1.20	1.68 ± 1.09	*∗∗∗*
Total cholesterol	4.23 ± 1.25	3.94 ± 1.08	*∗∗∗*
Adiposity			
Body mass index (kg/m^2^)	29.69 ± 5.66	29.44 ± 5.49	*∗*
Waist (cm)	102.97 ± 17.54	101.32 ± 15.56	*∗∗∗*
Exercise capacity (peak METs)	6.5 ± 2.8	7.2 ± 3.1	*∗∗∗*
Elevated depressive symptoms (%)	10%	4%	*∗∗*
Elevated anxiety symptoms (%)	3%	2%	

*Note*. ^*∗*^ *p* < .05, ^*∗∗*^ *p* < .01, ^*∗∗∗*^ *p* < .001 METs, metabolic equivalents. ^†^Paired *t*-tests.

## References

[1] Mendis S., Puska P., Norrving B. (2011). *Global Atlas on Cardiovascular Disease Prevention and Control*.

[2] Stone J. A., Arthur H. M., Arnold C. J. (2009). *Canadian Guidelines for Cardiac Rehabilitation and Cardiovascular Disease Prevention: Enhancing the Science, Refining the Art*.

[3] Heran B. S., Chen J. M., Ebrahim S. (2011). Exercise-based cardiac rehabilitation for coronary heart disease. *Cochrane Database of Systematic Reviews*.

[4] Grace S. L., Tan Y., Marcus L. (2012). Perceptions of cardiac rehabilitation patients, specialists and rehabilitation programs regarding cardiac rehabilitation wait times. *BMC Health Services Research*.

[5] Bajaj R. R., Goodman S. G., Yan R. T. (2013). Treatment and outcomes of patients with suspected acute coronary syndromes in relation to initial diagnostic impressions (insights from the Canadian global registry of acute coronary events [GRACE] and Canadian registry of acute coronary events [CANRACE]). *American Journal of Cardiology*.

[6] Ellison L. F. (2010). Measuring the effect of including multiple cancers in survival analyses using data from the Canadian Cancer Registry. *Cancer Epidemiology*.

[7] Fang J., Kapral M. K., Richards J., Robertson A., Stamplecoski M., Silver F. L. (2011). The registry of Canadian stroke network: an evolving methodology. *Acta Neurologica Taiwanica*.

[8] Swabey T., Suskin N., Arthur H. M., Ross J. (2004). The Ontario cardiac rehabilitation pilot project. *Canadian Journal of Cardiology*.

[9] CHHS-AP Steering Committee (2009). *The Canadian Heart Health Strategy and Action Plan: Building a Heart Healthy Canada*.

[10] Canadian Association of Cardiovascular Prevention and Rehabilitation CCRR Data Access Process. http://www.cacr.ca/resources/DAP.cfm.

[11] Bruce R. A., Kusumi F., Hosmer D. (1973). Maximal oxygen intake and nomographic assessment of functional aerobic impairment in cardiovascular disease. *American Heart Journal*.

[12] Anderson T. J., Grégoire J., Hegele R. A. (2013). 2012 update of the Canadian Cardiovascular Society guidelines for the diagnosis and treatment of dyslipidemia for the prevention of cardiovascular disease in the adult. *Canadian Journal of Cardiology*.

[13] Zigmond A. S., Snaith R. P. (1983). The hospital anxiety and depression scale. *Acta Psychiatrica Scandinavica*.

[14] Beck A. T., Ward C. H., Mendelson M., Mock J., Erbaugh J. (1961). An inventory for measuring depression. *Archives of general psychiatry*.

[15] Hays R. D., Sherbourne C. D., Mazel R. (1995). *User's Manual for the Medical Outcomes Study (MOS) Core Measures of Health-Related Quality of Life*.

[16] World Health Organization (1995). *Physical Status: The Use and Interpretation of Anthropometry*.

[17] Hypertension Canada (2014). *Canadian Hypertension Education Program Recommendations for Diagnosis*.

[18] Grundy S. M., Cleeman J. I., Daniels S. R. (2005). Diagnosis and management of the metabolic syndrome: an American Heart Association/National Heart, Lung, and Blood Institute scientific statement. *Circulation*.

[19] Dafoe W., Arthur H., Stokes H., Morrin L., Beaton L. (2006). Universal access: but when? Treating the right patient at the right time: access to cardiac rehabilitation. *Canadian Journal of Cardiology*.

[20] Brual J., Gravely-Witte S., Suskin N., Stewart D. E., Macpherson A., Grace S. L. (2010). Drive time to cardiac rehabilitation: at what point does it affect utilization?. *International Journal of Health Geographics*.

[21] Grace S. L., Poirier P., Norris C. M., Oakes G. H., Somanader D. S., Suskin N. (2014). Pan-Canadian development of cardiac rehabilitation and secondary prevention quality indicators. *Canadian Journal of Cardiology*.

[22] Collins C., Suskin N., Aggarwal S., Grace S. L. (2015). Cardiac rehabilitation wait times and relation to patient outcomes. *European Journal of Physical Medicine*.

[23] Grace S. L., Leung Y. W., Reid R., Oh P., Wu G., Alter D. A. (2012). The role of systematic inpatient cardiac rehabilitation referral in increasing equitable access and utilization. *Journal of Cardiopulmonary Rehabilitation and Prevention*.

[24] Samayoa L., Mola A., Terzic C., Thomas R., Grace S. L. (2014). Ethnocultural diversity in cardiac rehabilitation: a call to action. *Journal of Cardiopulmonary Rehabilitation & Prevention*.

[25] Perk J., De Backer G., Gohlke H. (2012). European guidelines on cardiovascular disease prevention in clinical practice (version 2012): the fifth joint task force of the European society of cardiology and other societies on cardiovascular disease prevention in clinical practice. *International Journal of Behavioral Medicine*.

[26] Ades P. A., Savage P. D., Harvey-Berino J. (2010). The treatment of obesity in cardiac rehabilitation. *Journal of Cardiopulmonary Rehabilitation and Prevention*.

[27] Savage P. D., Brochu M., Poehlman E. T., Ades P. A. (2003). Reduction in obesity and coronary risk factors after high caloric exercise training in overweight coronary patients. *American Heart Journal*.

[28] Kavanagh T., Mertens D. J., Hamm L. F. (2002). Prediction of long-term prognosis in 12 169 men referred for cardiac rehabilitation. *Circulation*.

[29] Myers J., Prakash M., Froelicher V., Do D., Partington S., Atwood J. E. (2002). Exercise capacity and mortality among men referred for exercise testing. *The New England Journal of Medicine*.

[30] Jolliffe J. A., Rees K., Taylor R. S., Thompson D., Oldridge N., Ebrahim S. (2001). Exercise-based rehabilitation for coronary heart disease. *Cochrane Database of Systematic Reviews*.

[31] Niebauer J., Mayr K., Harpf H. (2014). Long-term effects of outpatient cardiac rehabilitation in Austria: a nationwide registry. *Wiener Klinische Wochenschrift*.

[32] Faris P. D., Grant F. C., Galbraith P. D., Gong Y., Ghali W. A. (2004). Diagnostic cardiac catheterization and revascularization rates for coronary heart disease. *Canadian Journal of Cardiology*.

