# Supplementary material for: The Canadian Cardiac Rehabilitation Registry: Inaugural Report on the Status of Cardiac Rehabilitation in Canada
Source: Rehabil Res Pract. 2015 Aug 18;2015:278979. doi: 10.1155/2015/278979 (PMC4556335; doi:10.1155/2015/278979)
Supplement: Supplementary file 1 — The Supplementary Material consists of the data dictionary 1.0 for the Canadian Cardiac Rehabilitation Registry. This is a listing of all variables assessed in the registry, with their definition (i.e., data sources, time windows, units of measurement). There are variables which are captured at cardiac rehabilitation intake assessment only, as well as variables which are assessed at both the intake and discharge assessments. [file 278979.f1.pdf]

Canadian Cardiac Rehab Registry

By: CACRC

DATA ELEMENT DEFINITIONS

May 2012

Copyright © Canadian Association of Cardiac Rehabilitation 2012
To be used with permission only. Not for re-distribution.

Copyright © Canadian Association of Cardiac Rehabilitation 2012
To be used with permission only. Not for re-distribution

Table of Contents

GENERAL SUMMARY
Patient information 1
- Identifying information 1
- Secondary information 1

Cardiac Rehab Program 3

Service Referrals 8

Risk Factors 9

INTAKE/DISCHARGE 1
Measurements 12
Stress Test 16

INTAKE/DISCHARGE 2
Occupation 20
Smoking 22
Psycho-social 23

MEDICATIONS 25

Appendix A i

Copyright © CACR

Copyright © Canadian Association of Cardiac Rehabilitation 2012
To be used with permission only. Not for re-distribution

GENERAL SUMMARY
PATIENT INFORMATION
IDENTIFYING INFORMATION
Source Unique ID

This number is electronically generated, encrypted, and represents a unique identifier that indicates which clinical record this patient relates to.

Unique Patient ID

Enter the patient's permanent chart number ("patient ID number", "hospital number", "chart ID", "hospital ID") that corresponds to the patient's permanent unique file number. If you are unsure which is the permanent hospital file number ask a member of the Health Records Department staff which number is sent to the Canadian Institute of Health Information (CIHI). This is not a "visit" number. The CCRR automatically encrypts this number.

Data Source: Face sheet of the patient chart or on the Bradma plate stamped on the upper right corner of most pages of the chart.

Data Entry: The HID number is institution specific and ranges between 6 and 10 digits.

Year of Birth

Indicate the patient's year of birth.

Gender

Select the patient's sex.

Data Entry: Choices available are:

- Male
- Female
- Other

Consent Given

Confirm that patient consent was obtained for data collection and analysis.

Data Entry: Choices available are:

- Yes
- No

SECONDARY INFORMATION
Ethnicity

Refers to the ethnic or cultural group (s) to which the patient and/or their ancestors belong. An ancestor is someone from whom a person is descended and is usually more distant than a grandparent. Ancestry should not be confused with citizenship or nationality. Ethnic or cultural origin refers to the ethnic "roots" of ancestral background of the population. If multiple ethnic origins are indicated, please only report the **"paternal ancestry"**. Select only one from the list provided.

You may need to explain to the patient why this information is necessary. You may choose to say **"this information is collected to promote and ensure the equal opportunity for everyone to access cardiac rehabilitation services in Canada".** The

Copyright © CACR

information generated will ensure that future patients in Canada will have equal access to services.

Data Sources: Patient intake assessment/interview.

Data Entry: Indicate only one of the ethnic origins listed (list of ethnic origins based on the 2001 Canadian Census data from Statistics Canada).

- Aboriginal
- Arab/West Asian (i.e. Armenian, Egyptian, Iranian, Lebanese, Moroccan, Afganistan)
- Black
- Chinese
- Filipino
- Japanese
- Korean
- Latin American
- South Asian (i.e. Pakistani, East Indian, Bangladesh, Sri Lankan)
- South East Asian (i.e. Cambodian, Indonesian, Loatian, Vietnamese)
- White/Caucasian
- Other
- Unknown

Marital Status

Select the most appropriate response that corresponds with the patient's marital status.

Data Source: Face sheet of the patient chart, Admission history, History and Physical, Nursing Admission note, hospital information system, Intake Assessment

Data Entry: Choices available are:

- Single
- Married
- Divorced
- Widowed
- Common-Law Union
- Separated
- Unknown

Language Preference

Refers to the patient's preference to communicate in one of Canada's two official languages (English or French).

Data Entry: Choices available are:

- English
- French

Education (Highest Level Achieved)

Refers to the completion of secondary school or the highest level of university education achieved.

Data Source: Admission history, intake assessment for cardiac rehabilitation.

Data Entry:

- No High School – patient did no complete all high school requirements and no Secondary School Diploma was awarded (or equivalent).

Copyright © CACR

- High School – patient completed high school and Secondary School Diploma was awarded (or equivalent).
- Bachelor Degree – patient completed an undergraduate program from University and Bachelors Degree was awarded.
- Post-graduate degree – patient completed advanced preparation (i.e. MD, Master's degree, Ph.D., veterinary or dentistry).

Family Support

Select the most appropriate response that corresponds with the patient's living situation.
Data Source: Face sheet of the patient chart, Admission history, History and Physical, Nursing Admission note, hospital information system, Intake Assessment
Data Entry: Choices available are:

- Lives alone – patient is living alone.
- Lives with Spouse/Partner – patient is currently living with a spouse or partner (married or common-law)
- Lives with Friends/Family – patient is living with others that does not include a spouse or partner. This could include children, parents, in-laws.
- Unknown

Residence

Select the most appropriate response to categorize the patient's primary place of residence.
Data Source: Face sheet of the patient chart, Admission history, History and Physical, Nursing Admission note, hospital information system, Intake Assessment
Data Entry: Choices available are:

- Home/Apartment/Condominium
- Long-term Care Institution
- Rehabilitation Hospital
- Acute Care Hospital
- Unknown

Travel Time to Rehab

Indicate the estimated time taken for a single one-way trip from the patient's residence to the rehabilitation facility where the majority of the ongoing rehabilitation service will be provided. Travel time is independent of modality used.
Data Source: Cardiac rehabilitation intake interview/ assessment.
Data Entry: Choices available are:

- 0-30 minutes
- 31-45 minutes
- 46-60 minutes
- > 60 minutes
- Unknown

CARDIAC REHAB PROGRAM

Referral Event(s)

The referral event refers to the most recent event preceding the patient's referral to cardiac rehabilitation. Referral events are considered only those events occurring within

Copyright © CACR

the 2-year period prior to referral. The event does not necessarily require hospitalization.

There may be more than one possible referral event reported if the second referral event occurred within the same hospitalization period. The following are possible scenarios:

1. If the patient had coronary artery bypass graft surgery with an aortic valve replacement then both CABG and AVR would be considered referral events.
2. If the patient had a myocardial infarction and percutaneous transluminal coronary angioplasty within the same hospitalization period then both MI and PCI would be considered referral events.
3. If patient had an MI, was referred for cardiac rehabilitation and had another event during the period between referral and intake, the MI would count as the referral event and the secondary event (not within the same hospitalization period) would not be a referral event.

Data Source: Patient's chart, referral form, health professional referral letter, patient intake interview.

Data Entry: Choices available are:

- **Not Stable CAD / Angina**
- **Acute coronary syndrome (ACS) - Unspecified**
- **Acute coronary syndrome (ACS) - Myocardial infarction - Unspecified**
- **Acute coronary syndrome (ACS) - Myocardial infarction - STEMI**
- **Acute coronary syndrome (ACS) - Myocardial infarction - Non STEMI**
- **Acute coronary syndrome (ACS) - Unstable Angina**
- **Percutaneous Intervention (PCI) - Unspecified**
- **Percutaneous Intervention (PCI) - Drug Eluting Stent**
- **Percutaneous Intervention (PCI) - Bare Metal Stent**
- **Bypass Surgery (CABG)**
- **Valve Surgery - Unspecified**
- **Valve Surgery - Aortic**
- **Valve Surgery - Mitral**
- **Valve Surgery - Other**
- **Heart Failure - Unspecified**
- **Heart Failure - Cardiomyopathy**
- **Devices - Unspecified**
- **Devices - Pacemaker**
- **Devices - ICD**
- **Post Transplant**
- **Cerebral vascular disease - Unspecified**
- **Cerebral vascular disease - TIA**
- **Cerebral vascular disease - Stroke**
- **Perpheral Vascular disease**
- **High Risk Primary Prevention**
- **Unknown**

Referral Event(s) Date(s)

Enter the date(s) of the event(s). If there is more than one date as in scenario #2 above, enter a date for each event selected. However, the date corresponding to the most recent

Copyright © CACR

Copyright © Canadian Association of Cardiac Rehabilitation 2012
To be used with permission only. Not for re-distribution

referral event will be considered the Referral Event Date.
Data Source: Face sheet of the patient chart, hospital information system, nursing admission note, physician referral note, history and physical, or cardiac rehabilitation intake interview.

Referral Location

Where did the referral come from?
Data Source: Patient's chart
Data Entry: Choices available are:

- In-patient unit
- Outpatient [diagnostic testing/ambulatory clinic/MD office]
- Emergency room
- Cardiac diagnostics / Intervention
- Physician office
- Outpatient clinic
- Other
- Unknown

Referral Date

Enter the date the patient referral is made to the program (might not be the date the form is sent).
Data Source: Face sheet of the patient's chart, referral form at the CR program, others as indicated.

Referral Receipt Date

Enter the date the patient referral is received by the program.
Data Source: Face sheet of the patient's chart, referral form at the CR program, date received stamped or fax date on referral form, others as indicated.

Automatic Referral

Was this referral initiated automatically?
Data Entry: Choices available are:

- Yes
- No
- Unknown

Intake Visit Date

Enter the date the patient attended the first appointment at the CR program (this may be the intake assessment, rehabilitation exercise test, educational class, or other services)
Data Source: Patient's chart.

Date the CR Program Began

Enter the date the patient officially began the cardiac rehabilitation program. Please note that this applies to both on and off-site (home-based) cardiac rehabilitation programs.

Copyright © CACR

Date of CR Program Exit

Date of the patient's CR exit assessment. This refers to the patient's final contact with a CR professional where reporting of the final assessment and education might occur. Please note that it is possible for this to occur before or after the program discharge date.

Discharge Date

Indicate the date of discharge from the cardiac rehabilitation program. The discharge date could be based on the patient completing the CR month program, the patient completing all recommended services, or a date prior to completing the 6 month rehabilitation program due to specific reasons. This date should closely correspond to the patient's last documented rehabilitation visit.

Data Source: Patient's chart.

Work Return Date

Date that patient is actually able to return to work, if applicable.

CR Program Completion

Indicate if the patient completed the CR program.

Interim Event(s)

List any cardiac event(s) that occurred after referral to and prior to discharge from cardiac rehabilitation. Multiple post-referral events are possible.

Data Source: Patient's chart, physician progress note/assessment, nursing intake assessment note, hospitalization record(s), patient report (must follow-up with primary care physician to validate).

Data Entry: Choices available are:

- Stable CAD / Angina
- Acute coronary syndrome (ACS) – Unspecified
- Acute coronary syndrome (ACS) - Myocardial infarction – Unspecified
- Acute coronary syndrome (ACS) - Myocardial infarction – STEMI
- Acute coronary syndrome (ACS) - Myocardial infarction - Non STEMI
- Acute coronary syndrome (ACS) - Unstable Angina
- Percutaneous Intervention (PCI) – Unspecified
- Percutaneous Intervention (PCI) - Drug Eluting Stent
- Percutaneous Intervention (PCI) - Bare Metal Stent
- Bypass Surgery (CABG)
- Valve Surgery – Unspecified
- Valve Surgery – Aortic
- Valve Surgery – Mitral
- Valve Surgery – Other
- Heart Failure – Unspecified
- Heart Failure – Cardiomyopathy
- Devices – Unspecified
- Devices – Pacemaker
- Devices – ICD
- Post Transplant
- Cerebral vascular disease – Unspecified
- Cerebral vascular disease – TIA

Copyright © CACR

Copyright © Canadian Association of Cardiac Rehabilitation 2012
To be used with permission only. Not for re-distribution

- Cerebral vascular disease – Stroke
- Perpheral Vascular disease
- High Risk Primary Prevention
- Depression
- Death – Cardiac
- Death - Non Cardiac
- Unknown

Interim Event(s) Date(s)

Date(s) of cardiac event(s) occurring after referral to and prior to discharge from cardiac rehabilitation.

Data Source: Patient's chart, physician progress note/assessment, nursing intake assessment note, hospitalization record(s), patient report (must follow-up with primary care physician to validate).

Premature Termination(s)

Indicate the reason for premature termination of the patient's cardiac rehab program, if applicable

Data Entry: Choices available are:

- Lost to follow-up
- Patient choice (quit)
- Relocation
- Event – Cardiac
- Event – Other
- Death
- Unknown

Premature Termination(s) Date

If applicable, enter the date that patient prematurely terminated participation in the program.

Nutritional Sessions Prescribed

Enter the total number of nutritional counselling sessions scheduled for the patient's cardiac rehab program.

Nutritional Sessions Completed

Enter the total number of nutritional counselling sessions attended by the patient during their rehab program.

Psychological Sessions Prescribed

Enter the total number of psychological counselling sessions scheduled for the patient's cardiac rehab program.

Psychological Sessions Completed

Enter the total number of psychological counselling sessions attended by the patient during their rehab program.

Copyright © CACR

Copyright © Canadian Association of Cardiac Rehabilitation 2012
To be used with permission only. Not for re-distribution

Smoking Cessation Sessions Prescribed

Enter the total number of smoking cessation sessions scheduled for the patient's cardiac rehab program.

Smoking Cessation Sessions Completed

Enter the total number of smoking cessation sessions attended by the patient during their rehab program.

Vocational Counselling Sessions Prescribed

Enter the total number of vocational counselling sessions scheduled for the patient's cardiac rehab program.

Vocational Counselling Sessions Completed

Enter the total number of vocational counselling sessions attended by the patient during their rehab program.

Number of Exercise Sessions Prescribed

Enter the total number of exercise classes scheduled for the patient's cardiac rehab program.

Number of Exercise Sessions Completed

Enter the total number of exercise classes attended by the patient during their rehab program.

SERVICE REFERRALS

Information regarding the service referral data elements can most likely be found in the patient chart.

Except where stated otherwise, choices available for these data elements are as follows:

- **Offered and Accepted**
- **Offered and Declined**
- **Unknown**
- **Not Offered**

[N.B. If a patient does not accept or decline the offer please select 'Offered and Declined' .]

On-site exercise training: once per week

Was the patient offered or did they accept a once per week on-site exercise training program?

On-site exercise training: twice per week

Was the patient offered or did they accept a twice per week on-site exercise training program?

On-site exercise training: three or more times per week

Was the patient offered or did they accept a three or more times per week on-site exercise training program?

Copyright © CACR

Off-site exercise training: once per week

Was the patient offered or did they accept a once per week off-site training program?

Off-site exercise training: twice per week

Was the patient offered or did they accept a twice per week off-site training program?

Off-site exercise training: three or more times per week

Was the patient offered or did they accept a three or more per week off-site training program?

Nutrition 1:1 counsel

Was the patient offered or did they accept one on one nutritional counselling?

Smoking Cessation

Was the patient offered or did they accept assistance to quit smoking within the CR program?

- If patient is a non-smoker, please select 'Not offered'

Referral to Smoking Cessation Program

Was the patient referred to a smoking cessation program outside of the CR program?

Referral to Stress Management

Was the patient referred to a stress management program?

Referral to Depression Counselling

Was the patient referred to a depression counselling program?

Referral to Anxiety Counselling

Was the patient referred to an anxiety counselling program?

RISK FACTORS

Sedentary Lifestyle

According to the Canadian Association of Cardiac Rehabilitation physical inactivity (Sedentary Lifestyle) denotes a level of activity less than that needed to maintain good health. The minimum requirements for physical activity to maintain health, according to the 2009 CACR guidelines, is 30-60 minutes of moderate to vigorous physical activity most, preferally all, days of the week.

Data Sources: Initial intake visit, patient interview/questionnaire. Patient asked to describe their physical activity level prior to Cardiac Rehabilitation Intake.

Data Entry:

- **Yes -** if patient does not participate in moderate to vigorous physical activity for 30-60 minutes, most days of the week
- **No -** if patient participates in 30-60 minutes or more of moderate to vigorous exercise most days of the week
- **Unknown -** if the patient's physical activity cannot be determined by the sources defined or the reliability of the information is questionable.

Copyright © CACR

Hyperlipidemia

Is there a documented history of Hyperlipidemia?

Data Source: History & Physical, Emergency Department record/notes, Nursing Admission notes, Physician's progress notes, lab results (do not include blood work results within 8 weeks of an event e.g MI, CABG)

Data Entry:

- **Yes -** if there is a history of Hyperlipidemia, if patient is on lipid lowering medications and/or if LDL >2.0 mmol/L in patients with vascular disease (including CAD) or diabetes
- **No -** if there is no history of or treatment for Hyperlipidemia
- **Unknown -** if there is no mention of Hyperlipidemia in any of the recommended sources listed above.

Hypertension

Is there a documented history of Hypertension (HTN)? Any of the following documented terms apply:

- Accelerated Blood Pressure
- Benign hypertension
- Elevated blood pressure (BP)
- High blood pressure (BP)
- Hypertension (HTN)
- Hypertensive heart disease
- Labile hypertension
- Renal hypertension
- Renovascular hypertension
- Systemic hypertension
- Systolic hypertension
- Uncontrolled hypertension

Those conditions that should **NOT** be considered hypertension are:

- Intraocular hypertension
- Portal hypertension
- Pulmonary hypertension (any)

Data Source: History & Physical, Emergency Department record/notes, Nursing Admission notes, Physician's progress notes, current treatment for hypertension.

Data Entry:

- **Yes -** if there is a history of Hypertension.
- **No -** if there is no history of Hypertension
- **Unknown -** if there is no mention of Hypertension in any of the recommended sources listed above.

Smoking

Note: Smoking is defined as a minimum of 1 cigarette per day. The following should NOT be considered as 'smoking': Chewing tobacco, cigar smoking, marijuana, and pipe smoking.

Copyright © CACR

Copyright © Canadian Association of Cardiac Rehabilitation 2012
To be used with permission only. Not for re-distribution

Indicate if the patient has smoking as a risk factor.

Fam Hx of CAD

Was there a family history of early onset coronary artery disease?

Data Source: History & Physical, Emergency Department record/notes, Nursing Admission notes, Physician’s progress notes, patient intake assessment.

Data Entry: “Yes”, “No” or “Unknown (default).

- **Yes -** if there is a family history of coronary heart disease in a first degree relative (i.e. father, mother, brother or sister) with an early onset defined as mother/sister <65 years old and /or father/brother <55 years old in any of the recommended sources listed above.
- **No -** if there is a family history of coronary heart disease in a first degree relative (i.e. father, mother, brother or sister) but it has occurred outside of the age frames defined above.
- **No -** if there is no family history of coronary heart disease in a first-degree relative (i.e. father, mother, brother or sister).
- **Unknown** - if family history is not known

Diabetes

Was there a documented diagnosis of diabetes or treatment for diabetes? Any of the following documented terms apply:

- Adult Onset Diabetes Mellitus
- Diabetes
- Diabetes Mellitus (DM)
- Diet controlled diabetes
- DM Type I (1) or Type II (2)
- Drug induced diabetes
- Juvenile Diabetes
- Non-insulin dependent Diabetes Mellitus
- Any treatment for diabetes

Those conditions that should **NOT** be considered as diabetes are:

- Diabetes insipidus
- History of gestational diabetes
- Impaired fasting glucose
- Glucose intolerance

Data Source: History & Physical, Emergency Department record/notes, Nursing Admission notes, Physician’s progress notes, Intake Assessment, current treatment for diabetes.

Data Entry:

- **Yes** - if diabetes is mentioned in any of the recommended sources listed above.
- **No -** if there is no history of diabetes
- **Unknown -** if there is no mention of diabetes in any of the recommended sources listed above.

Copyright © CACR

Diabetes Type

If "Yes" is indicated above, enter:

- Type 1
- Type 2
- Unknown.

INTAKE/DISCHARGE 1

MEASUREMENTS

The following measures are to be completed at program intake and discharge.

CV STATUS

CCS Angina

Enter the corresponding Canadian Cardiovascular Society Angina Class at the time of intake and discharge. The CCS Angina Class must be determined post – referral event and should be assessed at the time of intake. If not assessed at intake and no post-event documentation is available, select Unknown from the pick list.

Data Source: Face sheet of the patient chart, Admission history, History and Physical, Nursing Admission note, Physician's progress notes, hospital information system, Discharge summary or angiography or echocardiography test reports.

Data Entry: Choices available are:

- **0** – Asymptomatic
- **1** - Ordinary physical activity such as walking or climbing does not cause angina. Angina with strenuous, rapid or prolonged exertion at work or recreation.
- **2** - Slight limitation of ordinary activity. Walking or climbing stairs rapidly, walking uphill, walking or climbing stairs after meals or in cold or in wind, or under emotional stress or during the few hours after wakening. Walking more than 2 blocks on the level and climbing more than one flight of stairs at a normal pace and in normal conditions.
- **3** - Marked limitation of ordinary physical activity. Walking one or two blocks on the level or climbing one flight of stairs in normal conditions and at a normal pace.
- **4** - Inability to carry out any physical activity without discomfort; angina may be present at rest
- **Unknown**

NYHA class

Enter a value from I to IV as indicated at intake and discharge. The NYHA Functional Class needs to be determined post-referral event and should be assessed at the time of intake. If not assessed at intake and no post-event documentation is available, select Unknown from the pick list.

Data Source: Face sheet of the patient chart, Admission history, History and Physical, Nursing Admission note, Physician's progress notes, hospital

Copyright © CACR

information system, Discharge summary or angiography or echocardiography test reports.
Data Entry: Choices available are:

- **I -** Patients with cardiac disease but without resulting limitations of physical activity. Ordinary physical activity does not cause undue fatigue, palpitation, dyspnea, or anginal pain
- **II -** Patients with cardiac disease that results in a slight limitation of physical activity. Patients are comfortable at rest, but ordinary physical activity results in fatigue, palpitation, dyspnea, or anginal pain
- **III -** Patients with cardiac disease that results in a marked limitation of physical activity. Patients are comfortable at rest, but less than ordinary physical activity results in fatigue, palpitation, dyspnea, or anginal pain
- **IV -** Patients with cardiac disease that results in an inability to carry on any physical activity without discomfort; fatigue, dyspnea, or anginal pain may be present; if any physical activity is undertaken, symptoms increase. Symptoms may be present at rest.
- **Unknown**

LV Function or LV Function Exact

Enter the exact left ventricular ejection fraction (LVEF), if available.

OR

If exact LVEF unavailable, enter the appropriate value from the pick list provided to indicate the patients LVEF or degree of left ventricular function. LV Function to be assessed post-referral event and/or within 6 months of Intake. If there is no available documentation, select Unknown from the pick list.

Data Source: Physician notes: Admission history, History and Physical, Admission note, Physician's progress notes, Discharge summary, or referral note; Primary source documentation: angiography or echocardiography test reports.

Data Entry: Select only one of the following that corresponds to the patients level of left ventricular function.

- Normal ≥50% or greater
- Mild 40% and 49%
- Moderate 30% and 39%
- Severe <30%
- Unknown

BIOCHEMISTRY

General Notes:

1. Blood work must be done under standard fasting conditions (9- 12 hours since last meal (Canadian Lipid Guidelines, 2006).
2. **Intake Values** - Values obtained within 1 month prior to and two weeks after intake are valid. Note that lipid results within 8 weeks of a cardiac event must be repeated to be valid. Blood work can be done on site or at a local lab. The CR program or referring physician can complete the requisition; whichever is the local practice.
3. **Discharge Values –** Values obtained within 1 month of discharge are valid.

Copyright © CACR

Copyright © Canadian Association of Cardiac Rehabilitation 2012
To be used with permission only. Not for re-distribution

Blood Sugar Profile

FBS (mmol/L)

This refers to the patients fasting blood glucose level.

Data Source: (1) Lab Report, (2) Emergency Department record/notes, (3) History & Physical, (4) Progress note.

Data Entry:

Enter in the space provided the value obtained for fasting glucose.

HBA1c (pct)

This refers to the patient's glycosylated hemoglobin level.

Data Source: (1) Lab Report, (2) Emergency Department record/notes, (3) History & Physical, (4) Progress note.

Data Entry:

Enter in the space provided the value obtained for glycosylated hemoglobin. This value should be entered as a decimal. For example, an HbA1c of 6% should be entered as 0.06.

Lipid Profile (mmol/L)

Total Cholesterol (TC) (mmol/L)

What was the patient's total cholesterol value.

Data Source: (1) Lab Report, (2) Emergency Department record/notes, (3) History & Physical, (4) Progress note.

Data Entry: Enter the value for total cholesterol in mmol/L

Triglycerides (TG) (mmol/L)

What was the serum triglycerides (TG) result?

Data Source: (1) Lab Report, (2) Progress note.

Data Entry: Enter value obtained for serum Triglycerides in mmol/L.

High Density Lipoprotein (HDLC) (mmol/L)

What was the HDL cholesterol result?

Data Source: (1) Lab Report, (2) Progress note.

Data Entry: Enter value obtained for HDL cholesterol (HDL) in mmol/L.

Low Density Lipoprotein (LDLC) (mmol/L)

What was the LDL cholesterol result?

Data Source: (1) Lab Report, (2) Progress note.

Data Entry: Enter value obtained for LDL cholesterol (LDL) in mmol/L.

TCHDL

What was the TC/HDL ratio?

Data Source: (1) Lab Report; (2) Progress note.

Data Entry: Enter value obtained from Total cholesterol / HDL ratio.

Other Biochemistry Results

Serum Creatinine

What was the serum creatinine result?

Copyright © CACR

Copyright © Canadian Association of Cardiac Rehabilitation 2012
To be used with permission only. Not for re-distribution

Data Source: (1) Lab Report, (2) Progress note.
Data Entry: Enter value obtained for serum creatinine in µmol/L.

Microalbuminuria

What was the microalbuminuria result?
Data Source: (1) Lab Report, (2) Progress note.
Data Entry: Enter value obtained from microalbuminuria test in mg/day

hs-CRP

What was the high-sensitivity C-reactive protein (hs-CRP) result?
Data Source: (1) Lab Report, (2) Progress note.
Data Entry: Enter value obtained from hs-CRP test in mg/L.

ANTHROPOMETRIC

Weight (KG)

Enter the patient's weight in kilograms.
Data Source: Direct measurement at the intake assessment.
Data entry: Enter the patient's weight to the nearest 10th of a kilogram (i.e. 78.0 kg).

Height (m)

Enter the patient's height in metres at the initial intake visit.
Data Source: Direct measurement at the intake assessment visit.
Data Entry: Enter the patient's height to the nearest cm. The number you enter will be three digits (e.g. 1.72).

Waist (cm)

Enter the patient's waist measurement in centimeters (cm).
Data Source: Direct measurement at the intake assessment.
Data entry: Enter value obtained from patient's waist measurement to the nearest centimeter.

HIP (cm)

Enter the patient's hip measurement in centimeters (cm).
Data Source: Direct measurement at the intake assessment.
Data entry: Enter value obtained from patient's hip measurement to the nearest centimeter.

HEMODYNAMIC

2009 Canadian Hypertension Education Program Recommended Technique for Measuring Blood Pressure
(http://hypertension.ca/chep/wp-content/uploads/2009/01/2009-chep-recommendations_d3.pdf)

Place the cuff so that the lower edge is 3 cm above the elbow crease and the bladder is centered over the brachial artery. **The patient should be resting comfortably for 5 minutes in the seated position with back support.** The arm should be bare and supported with the antecubital fossa at heart level, as a lower position will result in an erroneously higher SBP and DBP. **There should be no talking, and patients' legs should not be crossed. At least three measurements should be taken in the same arm with the patient in the same position. The first reading should be discarded and the latter two averaged.**

Copyright © CACR

Systolic Blood Pressure / Diastolic Blood Pressure (SBP / DBP) (mm)

Enter the patient's systolic blood pressure (mmHg) and the patient's diastolic blood pressure (mmHg).

Data Source: Direct measurement at the intake assessment.

Data entry: Enter value obtained from patient's SBP and DBP.

Pulse Rate (bpm)

Enter the patient's heart rate (bpm).

Data Source: Direct measurement at the intake assessment.

Data entry: Enter value obtained from patient's pulse measurement.

STRESS TEST DATA

The following measures are required at program intake and discharge.

NOTE

A standardized protocol such as the Ramp, Bruce or Modified Bruce is highly recommended. In the rare circumstance that a patient refuses to complete a stress test, a previously completed symptom-limited maximal stress test will be acceptable, providing it meets the following criteria:

- A symptom-limited maximal stress test using standardized protocol;
- Stress test completed (1) post-referral event and within 1 month of the intake date, and (2) post-discharge and the patient has been stabilized in the period following the test;

Stress Test Location

Where was the stress test performed?

Data Entry: Choices available are:

- On Site – by CR Team
- Off Site – by other MD
- Unknown

Stress Test Date

When was the stress test performed?

Data Entry: Please use the calendar to enter the date of the exercise stress test.

Resting SBP (mm)

What was the patient's resting systolic blood pressure during the exercise stress test?

Data Source: Exercise stress test reports.

Data Entry:

- If two BPs were recorded for the same time or it was taken in both arms enter the **highest systolic reading**.
- If a range is given, record the mid-value i.e. 120-130 record 125.
- If the systolic BP is "0" in the chart enter "0".

Peak SBP (mm)

What was the patient's peak systolic blood pressure during the exercise stress test?

Copyright © CACR

Data Source: Exercise stress test reports.

Data Entry:

- If two BPs were recorded for the same time or it was taken in both arms enter the **highest systolic reading**.
- If a range is given, record the mid-value i.e. 120-130 record 125
- If the systolic BP is "0" in the chart enter "0".

Resting DBP (mm)

What was the patient's resting diastolic blood pressure during the exercise stress test?

Data Source: Exercise stress test report.

Data Entry:

- If two BPs were recorded for the same time and have the same diastolic BP record the one with the highest diastolic value.
- If a range is given, record the mid-value i.e. 60 – 70 record 65.
- If the first diastolic BP is recorded in the chart as "p" for palpatation find the next BP with both systolic and diastolic values recorded numerically.

Peak DBP (mm)

What was the patient's peak diastolic blood pressure during the exercise stress test?

Data Source: Exercise stress test report.

Data Entry:

- If two BPs were recorded for the same time and have the same diastolic BP record the one with the highest diastolic value.
- If a range is given, record the mid-value i.e. 60 – 70 record 65.
- If the first diastolic BP is recorded in the chart as "p" for palpatation find the next BP with both systolic and diastolic values recorded numerically.

Resting Heart Rate (HR)

What was the patient's resting heart rate during the exercise stress test?

Data Source: Exercise stress test report.

Data Entry:

- Enter the resting heart rate (beats per minute) recorded on the electrocardiogram during the exercise test. In the event that the HR is not accurate on the cardiogram, a palpated HR (apical or radial) may be used.

Peak Heart Rate (HR)

What was the patient's peak heart rate during the exercise stress test?

Data Source: Exercise stress test report.

Data Entry:

- Enter the peak heart rate (beats per minute) recorded on the electrocardiogram during the exercise test. In the event that the HR is not accurate on the cardiogram, a palpated HR (apical or radial) may be used.

Copyright © CACR

Copyright © Canadian Association of Cardiac Rehabilitation 2012
To be used with permission only. Not for re-distribution

GXT Protocol

What was the GXT protocol used for the exercise stress test?

Data Source: Exercise stress test report.

Data Entry: Choice available are:

- Athletic
- Balke
- Bruce
- Bruce-Modified
- Ramp (includes slow, regular, fast)
- Cycle
- 6 min walk
- Ramp
- Naughton
- Other
- Unknown

Exercise Time

What was the duration of exercise during the exercise stress test?

Data Source: Exercise stress test report.

Data Entry: Enter the duration of exercise in minutes. For example, a duration of 14 minutes and 30 seconds would be entered as 14.5.

Reason for Premature Termination

If the exercise stress test was terminated, provide the reason why.

Data Source: Exercise stress test report.

Data Entry: Choices available are:

- Fatigue/leg pain
- Dyspnea
- Angina
- ST changes
- Arrhythmias
- Drop in BP
- Hypertensive response
- Presyncopal
- Patient request
- Equipment problems
- MD instruction
- Other
- Unknown

Angina

Indicate if patient had documented angina during the exercise stress test.

Data Source: Exercise stress test report and or exercise test ECG

Data Entry: Choices available are:

- None
- Non-limiting
- Stopped Test

Copyright © CACR

- Not documented
- Unknown

Borg 10 Scale

If the Borg 10 Scale was used, enter the maximum Borg rating achieved at completion of the exercise stress test. The Borg rating is used to assess perceived level of exertion.

Data Source: Exercise stress test report.

Data Entry:

- Value from 0-10

10-Grade Scale	
**0**	Nothing
**0.5**	Very, very weak
**1**	Very weak
**2**	Weak (light)
**3**	Moderate
**4**	Somewhat strong
**5**	Strong (heavy)
**6**	
**7**	Very strong
**8**	
**9**	
**10**	Very, very strong

Borg 15 Scale

If the Borg 10 Scale was used, enter the maximum Borg rating achieved at completion of the exercise stress test. The Borg rating is used to assess perceived level of exertion.

Data Source: Exercise stress test report.

Data Entry:

- Value from 6-20

15-Grade scale	
**6**	
**7**	Very, very light
**8**	
**9**	Very light
**10**	
**11**	Fairly light
**12**	
**13**	Somewhat hard
**14**	
**15**	Hard
**16**	
**17**	Very hard
**18**	
**19**	Very, very hard
**20**	

Copyright © CACR

Copyright © Canadian Association of Cardiac Rehabilitation 2012
To be used with permission only. Not for re-distribution

Max ST Depression

Indicate the maximum level of ST segment depression. ST depression is defined as horizontal or down-sloping ST depression measured at 60 or 80ms after the J point.

Data Source: Physician interpretation on the exercise stress test report and/or exercise test ECG.

Data Entry: None or write in number of millimeters noted on the test.

Peak METs

Indicate the peak METs achieved during the test in the space provided. The METs can be estimated from standard equations using speed and grade, or can be calculated from the direct measurement of oxygen consumption using gas analysis.

Data Source: Exercise stress test report.

Data Entry: Enter the numeric value of the peak METs as indicated by the exercise stress test report to the nearest 1/10 of a MET (i.e. 5.4 METs).

Duke Treadmill Score

What was the Duke Treadmill Score (DTS)? The DTS is a weighted index combining ST segment deviation (depression or elevation), treadmill time and exercise-induced angina. It provides accurate diagnostic and prognostic information.

DTS = Exercise Time (regular Bruce protocol minutes)* – (5x ST deviation) – (4x Angina index)

Angina Index: 0=none, 1=during test, and 2=angina stopped test. Distinction between exercise-induced angina and non-anginal chest pain is based on the clinical judgement of the physician supervising the stress test. Emphasis is placed on reproducing the patient's usual presenting symptoms and the classic features of typical angina.

Data Source: Exercise stress test report

Data Entry: Enter value between –25 (highest risk) and +15 (lowest risk)

*Can be "back-calculated" from non-Bruce protocols solving for METs.

METs Directly Measured

Indicate if METs level was directly measured using gas analysis or estimated.

Data Source: Exercise stress test report.

Data Entry:

- **Yes -** if METs were directly measured by oxygen uptake
- **No -** if METs were estimated using standard equations from final workload level completed.

INTAKE/DISCHARGE 2

OCCUPATION

The following measures are recommended at program intake and discharge.

Occupation

Select the most appropriate response to describe the patient's most recent occupation prior to the intake appointment and at discharge from cardiac rehabilitation. The Occupation Types are the Porter-Pineo McRobert's classification based on the 1981

Copyright © CACR

Canadian Classification and Dictionary of Operations and can be assigned Socioeconomic Status using the Blishen Method.
Data Source: Admission history, History and Physical, Consultant's notes, Nursing Admission note, Intake Assessment.
Data Entry: Available choices are:

- **Self-Employed Professional -** patient is currently self-employed.
- **Semiskilled Manual**
- **Employed professional -** patient is currently employed outside of the home as a professional (ie. MD,PhD)
- **Foreman**
- **Skilled Clerical/Sales/Services**
- **Farm worker**
- **High-Level Manager** if the patient is currently employed in a managerial position.
- **Skilled Blue Collar**
- **Semiskilled Clerical/Sales/Services**
- **Farmer (owner)**
- **Semi-professional**
- **Technician**
- **Unskilled Clerical/Sales/Services**
- **Unskilled manual**
- **Middle-level manager**
- **Supervisor**
- **Homemaker** - patient is functioning as a homemaker in his or her home.
- **Not applicable**
- **Unknown**

Employment Status

Select the most appropriate response to describe the patient's employment status at the time of intake to and discharge from cardiac rehabilitation.
Data Source: Admission history, History and Physical, Consultant's notes, Nursing Admission note, Intake Assessment.
Data Entry: Available choices are:

- **Full-time -** patient is currently employed or self-employed outside the home (35 or greater hours per week).
- **Part-time -** patient is currently employed less than full-time outside the home (less than 35 hours per week).
- **Unemployed -** patient is temporarily laid off or unemployed and looking for work.
- **Retired -** patient is no longer working and not looking for employment.
- **Not/Never Employed Outside the Home -** patient is not working outside the home and is not looking for employment.
- **Short-term Disability -** patient is not currently working due to his/her illness and is expected to return to work in the near future.
- **Long-term Disability -** patient is not currently working due to his/her illness and is not expected to return to work in the near future.

Copyright © CACR

- **Employed - Permanent Restrictions -** patient is currently employed and defined as restricted duties due to the severity of his/her illness.
- **Employed – Modified Duties –** patient is currently employed and defined as modified duties due to the severity of his/her illness
- **Unknown**

Desired Occupation Status

Select the most appropriate response to describe the patient's desired occupation status prior to the intake appointment and at discharge from cardiac rehabilitation.

Data Source: Admission history, History and Physical, Consultant's notes, Nursing Admission note, Intake Assessment.

Data Entry: See list provided under 'Occupation'

Desired Occupation Status Achieved

Was the patient's desired occupational status achieved by discharge from cardiac rehabilitation?

Data Source: Discharge assessment/interview, patient's chart.

Data Entry: Indicate:

- **Yes** - patient achieved his/her desired occupation status by discharge
- **No** - patient did not achieve his/her desired occupation status by discharge

SMOKING

The following measures are recommended at program intake and discharge.

Note: Smoking is defined as a minimum of 1 cigarette per day. The following should NOT be considered as 'smoking': Chewing tobacco, cigar smoking, marijuana, and pipe smoking.

At the present time, do you smoke cigarettes?

Does the patient smoke at the present time (intake and discharge)?

Data Entry: Choices available are:

- Every Day
- Occasionally
- Not at all
- Unknown

When was the last time you smoked at all, even just one puff?

If patient is or was a smoker, indicate the last date that the patient smoked. **If conflicting information is documented, take the "worst" case.**

Data Source: History & Physical, Nursing Admission notes, Physician's progress notes, Intake Assessment.

Have you ever smoked cigarettes daily?

Indicate if the patient ever smoked cigarettes daily**.**

Data Entry: Choices available are:

- Yes
- No

Copyright © Canadian Association of Cardiac Rehabilitation 2012
To be used with permission only. Not for re-distribution

Copyright © CACR

At what age did you begin to smoke cigarettes every day?
Indicate the age that the patient began to smoke cigarettes daily.

On those days you smoked, how many cigarettes did you usually smoke?
Indicate the number of cigarettes smoked per day.

CO Level (if available)
If available, indicate the CO level (0-200 ppm).

CO Level Measuring Device
Indicate the device used to measure CO level.
Data Entry: Type in the name of the device.

NOTE: If you click, "Copy Intake Values to Discharge", the exact same information will populate the Discharge smoking data. This is useful for smoking data that does not change from intake to discharge from the CR program.

PSYCHO-SOCIAL

The following measures are recommended at program intake and discharge.

ANXIETY

Measure Performed
Was an anxiety measure performed?
Data Entry: Choices available are:

- Yes
- No

Score
What was the score of the anxiety measure?
Data Entry: Enter the numerical value.

At or above cutoff
Was this score at or above the cutoff for anxiety?
Data Entry: Choices available are:

- Yes
- No

Measure
What tool was used to measure anxiety?
Data Entry: Choices available are:

- **Anxiety & Depression Scale Anxiety Score (HADS-A)**
- **Beck Anxiety Inventory (BAI)**
- **Other, please specify**

Other measure
If "Other, please specify" was chosen above, please type the name of the measure in here.

DEPRESSION

Measure Performed
Was a depression measure performed?

Copyright © CACR

Data Entry: Choices available are:

- Yes
- No

Measure Score

What was the score of the depression measure?

Data Entry: Enter the numerical value.

At or above cutoff

Was this score at or above the cutoff for depression?

Data Entry: Choices available are:

- Yes
- No

Measure

What tool was used to measure depression?

Data Entry: Choices available are:

- **Anxiety & Depression Scale Depression Score (HADS-D)**
- **Beck Depression Inventory (BDI)**
- **Other, please specify**

Other measure

If "Other, please specify" was chosen above, please type the name of the measure in here.

QUALITY OF LIFE – PHYSICAL

Measure Performed

Was a physical QOL measure performed?

Data Entry: Choices available are:

- Yes
- No

Score

What was the score of the physical QOL measure?

Data Entry: Enter the numerical value.

Measure

What tool was used to measure physical QOL?

Data Entry: Choices available are:

- **SF-12**
- **SF-36**
- **Other, please specify**

Other measure

If "Other, please specify" was chosen above, please type the name of the measure in here.

QUALITY OF LIFE – MENTAL

Mental Performed

Was a mental QOL measure performed?

Data Entry: Choices available are:

- Yes
- No

Score

Copyright © CACR

What was the score of the mental QOL measure?
Data Entry: Enter the numerical value.

Measure

What tool was used to measure mental QOL?
Data Entry: Choices available are the same as for QOL - physical

Other measure

If "Other, please specify" was chosen above, please type the name of the measure in here.

QUALITY OF LIFE – OTHER

Measure Performed

Was another QOL measure performed?
Data Entry: Choices available are:

- Yes
- No

Score

What was the score of the other QOL measure?
Data Entry: Enter the numerical value.

Measure

What tool was used to measure other QOL?
Data Entry: Choices available are the same as for QOL - physical

Other measure

If "Other, please specify" was chosen above, please type the name of the measure in here.

MEDICATIONS

Enter all prescribed **cardiovascular-related** medications at intake and discharge from the CR program. Discharge medications could be those documented at the time of the exit **Stress Test** or during the **Exit Assessment.**

Class

Indicate the therapeutic class of the medication(s) noted above.
Data Entry: Please see Appendix A for the list of medications and therapeutic classes.

Name

Choose the generic and brand name of all cardiovascular-realted medications the patient is prescribed at intake and discharge.
Data Entry: Please see Appendix A for the list of medications and therapeutic classes.

Contraindicated

Is this medication contraindicated?
Data Entry: Choices available are:

- **Yes**
- **No**

Dose, units

- Indicate the doses of all noted medications.

Copyright © CACR

Data Entry: Enter numeric value.

- Indicate the medication unit(s) of the dose(s).
  Data Entry: Choices available are:
    - **Unknown**
    - **mg**
    - **ug**
    - **g**
    - **U**
    - **mL**
    - **mg/hr**
    - **packet**
    - **mg/day**
    - **ug/day**

Frequency

- Indicate the frequency that the patient was prescribed to take the medication(s).
  Data Entry: Enter the number of times per day that patient was prescribed to take the medication(s).
- Indicate the medication schedule (frequency and/or time of day).
  Data Entry: Choices available are:
    - **Unknown**
    - **od**
    - **qam**
    - **ac breakfast**
    - **ac lunch**
    - **qpm**
    - **ac dinner**
    - **qhs**
    - **bid**
    - **tid**
    - **qid**
    - **tid on a qid schedule**
    - **on in am, off at bedtime**
    - **q 5min x 3 prn**
    - **od prn**
    - **as needed (max 20 pieces/day)**
    - **as needed (max 80 sprays/day)**
    - **as needed (6-16 cartridges/day)**
    - **q4h prn**

NOTE: By clicking "»" next to each medication on the Medication home screen, the exact same information will populate the Discharge medication list. This is useful for medications and dosages that do not change from intake to discharge from the CR program.

Copyright © CACR

Copyright © Canadian Association of Cardiac Rehabilitation 2012
To be used with permission only. Not for re-distribution

APPENDIX A

TherapeuticClass	Generic	Brand
**analgesic combinations**	acetaminophen-tramadol 37.5 mg	Tramacet
analgesic combinations	acetylsalicylic acid-butalbital-caffeine	Tecnal
**angiotensin converting enzyme inhibitors**	benazepril	Lotensin
angiotensin converting enzyme inhibitors	captopril	captopril
angiotensin converting enzyme inhibitors	captopril	Novo-Captoril
angiotensin converting enzyme inhibitors	captopril	Apo-Capto
angiotensin converting enzyme inhibitors	cilazapril	Inhibace
angiotensin converting enzyme inhibitors	enalapril	enalapril
angiotensin converting enzyme inhibitors	enalapril	Apo-Enalapril
angiotensin converting enzyme inhibitors	enalapril	Vasotec
angiotensin converting enzyme inhibitors	enalaprilat	Vasotec
angiotensin converting enzyme inhibitors	fosinopril	Monopril
angiotensin converting enzyme inhibitors	lisinopril	Zestril
angiotensin converting enzyme inhibitors	perindopril	Coversyl
angiotensin converting enzyme inhibitors	quinapril	Accupril
angiotensin converting enzyme inhibitors	ramipril	Altace
angiotensin converting enzyme inhibitors	trandolapril	Mavik

Copyright © CACR

Copyright © Canadian Association of Cardiac Rehabilitation 2012
To be used with permission only. Not for re-distribution

**angiotensin II inhibitors**	candesartan study	DC-AHS-0006
angiotensin II inhibitors	candesartan	Atacand
angiotensin II inhibitors	candesartan or placebo study	DC-AHS-0006
angiotensin II inhibitors	eprosartan	Teveten
angiotensin II inhibitors	irbesartan	Avapro
angiotensin II inhibitors	losartan study	-
angiotensin II inhibitors	losartan	losartan
angiotensin II inhibitors	losartan	Cozaar
angiotensin II inhibitors	telmisartan	Micardis
angiotensin II inhibitors	valsartan	Diovan
**anorectal preparations**	hydrocortisone 1%-pramoxine 1%	Pramox HC
anorectal preparations	hydrocortisone 1%-pramoxine 1%	Proctofoam HC
anorectal preparations	zinc sulfate 0.5%	Anusol Ointment
anorectal preparations	zinc sulfate 0.5%-pramoxine 1%	Anusol Plus
anorectal preparations	zinc sulfate-pramoxine	Anusol Plus
anorectal preparations	zinc sulfate-hydrocortisone-pramoxine	Sab Anuzinc HC Plus
anorectal preparations	zinc sulfate-hydrocortisone-pramoxine	Proctodan HC
**antiadrenergic agents, centrally acting**	clonidine	Dixarit
antiadrenergic agents, centrally acting	clonidine	Novo-Clonidine
antiadrenergic agents, centrally acting	clonidine	clonidine
antiadrenergic agents, centrally acting	clonidine or placebo study	clonidine or placebo
antiadrenergic agents, centrally acting	clonidine transdermal or placebo study	clonidine or placebo
antiadrenergic agents, centrally acting	methyldopa	Apo-Methyldopa
antiadrenergic agents, centrally acting	methyldopa	methyldopa
**antiadrenergic agents, peripherally acting**	alfuzosin ER	Xatral
antiadrenergic agents, peripherally acting	doxazosin	Cardura
antiadrenergic agents, peripherally acting	prazosin	Novo-Prazin
antiadrenergic agents, peripherally acting	reserpine	Novo-Reserpine

Copyright © CACR

antiadrenergic agents, peripherally acting	tamsulosin CR	Flomax CR
antiadrenergic agents, peripherally acting	tamsulosin SR	Flomax
antiadrenergic agents, peripherally acting	terazosin	Apo-Terazosin
**antianginal agents**	amyl nitrite	Amyl Nitrite
antianginal agents	isosorbide dinitrate	Novo-Sorbide
antianginal agents	isosorbide dinitrate SR	Cedocard-SR
antianginal agents	isosorbide dinitrate SL	Apo-ISDN
antianginal agents	isosorbide mononitrate ER	Imdur
antianginal agents	nitroglycerin	Nitro-Dur
antianginal agents	nitroglycerin	Minitran
antianginal agents	nitroglycerin (0.4 mg/spray)	Nitrolingual
antianginal agents	nitroglycerin 2%	Nitrol
antianginal agents	nitroglycerin	Nitroglycerin
**antiarrhythmic agents**	adenosine	Adenocard
antiarrhythmic agents	amiodarone	Cordarone
antiarrhythmic agents	bretylium	Bretylium Tosylate
antiarrhythmic agents	disopyramide	disopyramide
antiarrhythmic agents	disopyramide	Rythmodan
antiarrhythmic agents	disopyramide ER	Norpace CR
antiarrhythmic agents	disopyramide	Rythmodan-LA
antiarrhythmic agents	dofetilide	Tikasyn
antiarrhythmic agents	flecainide	Tambocor
antiarrhythmic agents	flecainide	flecainide
antiarrhythmic agents	GAP-486 or placebo study	GAP-486 or placebo study
antiarrhythmic agents	ibutilide	Corvert
antiarrhythmic agents	lidocaine 0.5%	Xylocaine HCl
antiarrhythmic agents	lidocaine 1%	Xylocaine HCl
antiarrhythmic agents	lidocaine 1%	Lidocaine Hydrochloride
antiarrhythmic agents	lidocaine 1.5%-epinephrine 1:200000	Xylocaine HCl with Epinephrine
antiarrhythmic agents	lidocaine	Xylocard

Copyright © CACR

antiarrhythmic agents	lidocaine	Dextrose-Lidocaine Hydrochloride
antiarrhythmic agents	lidocaine 2%	Lidocaine Hydrochloride
antiarrhythmic agents	lidocaine 2%	Xylocaine HCl
antiarrhythmic agents	lidocaine 2%-epinephrine 1:100000	Xylocaine HCl with Epinephrine
antiarrhythmic agents	lidocaine 4%	Xylocaine Sterile
antiarrhythmic agents	mepivacaine 1%	Carbocaine HCl
antiarrhythmic agents	mexiletine	Mexitil
antiarrhythmic agents	procainamide	Procainamide Hydrochloride
antiarrhythmic agents	procainamide	procainamide
antiarrhythmic agents	procainamide SR	Procan SR
antiarrhythmic agents	procainamide SR	Pronestyl-SR
antiarrhythmic agents	propafenone	Rythmol
antiarrhythmic agents	quiniDINE	Quinidine Sulfate
antiarrhythmic agents	spinal tray with lidocaine	spinal tray with lidocaine
**anticoagulants**	warfarin to be ordered	warfarin to be ordered
**antihypertensive combinations**	amiloride-hydrochlorothiazide	Moduret
antihypertensive combinations	spironolactone-hydrochlorothiazide	Novo-Spirozine
antihypertensive combinations	spironolactone-hydrochlorothiazide	spironolactone-hctz
antihypertensive combinations	triamterene-hydrochlorothiazide	Apo-Triazide
**bile acid sequestrants**	cholestyramine light	PMS Cholestyramine Light
bile acid sequestrants	colestipol	Colestid
**calcium channel blocking agents**	amlodipine	amlodipine
calcium channel blocking agents	amlodipine	Norvasc
calcium channel blocking agents	diltiazem CD	Ratio-Diltiazem CD
calcium channel blocking agents	diltiazem ER	Tiazac
calcium channel blocking agents	diltiazem ER	Tiazac XC
calcium channel blocking agents	diltiazem SR	Cardizem SR
calcium channel blocking agents	diltiazem	Novo-Diltazem
calcium channel blocking agents	diltiazem CD	Cardizem CD
calcium channel blocking agents	diltiazem	Diltiazem Hydrochloride
calcium channel blocking agents	diltiazem	diltiazem
calcium channel blocking agents	felodipine ER	Plendil

Copyright © CACR

Copyright © Canadian Association of Cardiac Rehabilitation 2012
To be used with permission only. Not for re-distribution

calcium channel blocking agents	NIFEdipine	PMS NIFEdipine
calcium channel blocking agents	NIFEdipine PA	Apo-Nifed PA
calcium channel blocking agents	NIFEdipine ER	Adalat XL
calcium channel blocking agents	nimodipine	Nimotop
calcium channel blocking agents	verapamil SR	Isoptin SR
calcium channel blocking agents	verapamil	Gen-Verapamil
calcium channel blocking agents	verapamil ER	Chronovera
calcium channel blocking agents	verapamil	Verapamil Hydrochloride
calcium channel blocking agents	verapamil	verapamil
calcium channel blocking agents	verapamil	Novo-Veramil
**cardioselective beta blockers**	acebutolol	Monitan
cardioselective beta blockers	atenolol	Novo-Atenol
cardioselective beta blockers	atenolol	PMS-Atenolol
cardioselective beta blockers	atenolol	atenolol
cardioselective beta blockers	bisoprolol	Monocor
cardioselective beta blockers	esmolol	Brevibloc
cardioselective beta blockers	esmolol	esmolol 2500 mg/250 mL
cardioselective beta blockers	metoprolol	metoprolol
cardioselective beta blockers	metoprolol SR	Lopresor SR
cardioselective beta blockers	metoprolol	Apo-Metoprolol
cardioselective beta blockers	metoprolol	PMS-Metoprolol-L
cardioselective beta blockers	metoprolol	Betaloc
cardioselective beta blockers	metoprolol	Novo-Metoprol Uncoated
**cox-2 inhibitors**	celecoxib	Celebrex
cox-2 inhibitors	meloxicam	Mobicox
**fibric acid derivatives**	bezafibrate	Bezalip
fibric acid derivatives	bezafibrate SR	Bezalip
fibric acid derivatives	clofibrate	Novo-Fibrate
fibric acid derivatives	fenofibrate	Apo-Fenofibrate
fibric acid derivatives	fenofibrate MC	Lipidil Supra
fibric acid derivatives	fenofibrate MCR	Gen-Fenofibrate Micro
fibric acid derivatives	fenofibrate MCR	Apo-Feno-Micro

Copyright © CACR

Copyright © Canadian Association of Cardiac Rehabilitation 2012
To be used with permission only. Not for re-distribution

fibric acid derivatives	gemfibrozil	PMS Gemfibrozil
**herbal products**	ubiquinone	Coenzyme Co Q10
**HMG-CoA reductase inhibitors**	atorvastatin	Lipitor
HMG-CoA reductase inhibitors	fluvastatin	Lescol
HMG-CoA reductase inhibitors	lovastatin	Apo-Lovastatin
HMG-CoA reductase inhibitors	pravastatin	Pravachol
HMG-CoA reductase inhibitors	rosuvastatin	Crestor
HMG-CoA reductase inhibitors	rosuvastatin or placebo study	Aurora
HMG-CoA reductase inhibitors	simvastatin	Zocor
HMG-CoA reductase inhibitors	simvastatin	Apo-Simvastatin
**impotence agents**	alprostadil	Prostin Vr Sterile
impotence agents	sildenafil	Viagra
**inotropic agents**	digoxin	Lanoxin
inotropic agents	DOBUTamine	Dobutamine Hydrochloride
inotropic agents	DOPamine	Intropin
inotropic agents	DOPamine	Dopamine HCl In Dextrose
inotropic agents	milrinone	Primacor
inotropic agents	milrinone or placebo study	milrinone
**loop diuretics**	bumetanide	Burinex
loop diuretics	ethacrynic acid	Edecrin
loop diuretics	ethacrynic acid	Sodium Edecrin
loop diuretics	furosemide	Apo-Furosemide
loop diuretics	furosemide	Lasix
loop diuretics	furosemide	Furosemide
**miscellaneous antihyperlipidemic agents**	ezetimibe	Ezetrol
**non-cardioselective beta blockers**	carvedilol	Coreg
non-cardioselective beta blockers	labetalol	Apo-Labetalol
non-cardioselective beta blockers	labetalol	Trandate
non-cardioselective beta blockers	labetalol	labetalol
non-cardioselective beta blockers	nadolol	nadolol
non-cardioselective beta blockers	nadolol	Apo-Nadol
non-cardioselective beta blockers	oxprenolol	Trasicor

Copyright © CACR

Copyright © Canadian Association of Cardiac Rehabilitation 2012
To be used with permission only. Not for re-distribution

non-cardioselective beta blockers	oxprenolol SR	Slow Trasicor
non-cardioselective beta blockers	pindolol	Novo-Pindol
non-cardioselective beta blockers	propranolol	Inderal
non-cardioselective beta blockers	propranolol	propranolol
non-cardioselective beta blockers	propranolol	Novo-Pranol
non-cardioselective beta blockers	propranolol LA	Inderal
non-cardioselective beta blockers	sotalol	Sotacor
non-cardioselective beta blockers	sotalol	sotalol
non-cardioselective beta blockers	timolol	Apo-Timol
**platelet aggregation inhibitors**	AZD6140 or placebo study	AZD6140 or placebo 90 mg TAB
platelet aggregation inhibitors	clopidogrel	Plavix
platelet aggregation inhibitors	clopidogrel or placebo study	clopidogrel or placebo study
platelet aggregation inhibitors	clopidogrel or placebo study	Plavix
platelet aggregation inhibitors	clopidogrel or placebo study	clopidogrel or placebo
platelet aggregation inhibitors	CS 747 or clopidogrel study	Triton TIMI-38 study
platelet aggregation inhibitors	dipyridamole	dipyridamole
platelet aggregation inhibitors	dipyridamole	Apo-Dipyridamole
platelet aggregation inhibitors	dipyridamole	Novo-Dipiradol
platelet aggregation inhibitors	dipyridamole	Persantine
platelet aggregation inhibitors	ticlopidine	Novo-Ticlopidine
**potassium-sparing diuretics**	amiloride	amiloride
potassium-sparing diuretics	amiloride	Midamor
potassium-sparing diuretics	spironolactone	Novo-Spiroton
potassium-sparing diuretics	spironolactone	spironolactone
**salicylates**	acetylsalicylic acid	ASA Suppositories
salicylates	acetylsalicylic acid EC	Novasen
salicylates	acetylsalicylic acid	ASA
salicylates	acetylsalicylic acid CHEW	Aspirin
salicylates	acetylsalicylic acid EC	Asaphen EC
salicylates	acetylsalicylic acid EC	Asaphen
salicylates	diflunisal	Apo-Diflunisal
**SSRI antidepressants**	citalopram	Celexa

Copyright © CACR

Copyright © Canadian Association of Cardiac Rehabilitation 2012
To be used with permission only. Not for re-distribution

SSRI antidepressants	fluoxetine	Apo-Fluoxetine
SSRI antidepressants	fluoxetine	Prozac
SSRI antidepressants	fluvoxamine	Luvox
SSRI antidepressants	paroxetine	Paxil
SSRI antidepressants	paroxetine CR	Paxil CR
SSRI antidepressants	sertraline	Novo-Sertaline
**sterile irrigating solutions**	sterile water	Bacteriostatic Water For Injection - 30 mL
sterile irrigating solutions	sterile water	Sterile Water for Irrigation
sterile irrigating solutions	sterile water	Sterile Water for Injection - 1 L
sterile irrigating solutions	sterile water	Sterile Water for Injection - 10 mL
sterile irrigating solutions	sterile water qs SYRINGE	Sterile Water for Injection
**sulfonamides**	sulfasalazine EC	PMS Sulfasalazine-E C
sulfonamides	sulfasalazine	PMS Sulfasalazine
sulfonylureas	gliclazide MR	Diamicron MR
**thiazide diuretics**	chlorthalidone	Novo-Thalidone
thiazide diuretics	hydrochlorothiazide	hydrochlorothiazide
thiazide diuretics	hydrochlorothiazide	PMS-Hydrochlorothiazide
thiazide diuretics	hydrochlorothiazide	Novo-Hydrazide
thiazide diuretics	hydrochlorothiazide	Apo-Hydro
thiazide diuretics	indapamide	Indapamide
thiazide diuretics	indapamide	Gen-Indapamide
thiazide diuretics	metolazone	Zaroxolyn
**thiazolidinediones**	pioglitazone	Actos
thiazolidinediones	rosiglitazone	Avandia
**tricyclic antidepressants**	amitriptyline	Novo-Triptyn
tricyclic antidepressants	clomiPRAMINE	Gen-Clomipramine
tricyclic antidepressants	clomiPRAMINE	Apo-Clomipramine
tricyclic antidepressants	desipramine	PMS Desipramine
tricyclic antidepressants	desipramine	Apo-Desipramine
tricyclic antidepressants	desipramine	Ratio-Desipramine Hydrochloride
tricyclic antidepressants	imipramine	Apo-Imipramine
tricyclic antidepressants	imipramine	imipramine

Copyright © CACR

tricyclic antidepressants	nortriptyline	Aventyl HCl
tricyclic antidepressants	nortriptyline	PMS Nortriptyline
tricyclic antidepressants	trimipramine	Novo-Tripramine
tricyclic antidepressants	trimipramine	Apo-Trimipramine
**vasodilators**	hydrALAZINE	Apo-Hydralazine
vasodilators	hydrALAZINE	Apresoline
vasodilators	hydrALAZINE	Novo-Hylazin
vasodilators	minoxidil	Loniten
**vitamins**	alfacalcidol	One-Alpha
vitamins	ascorbic acid	Apo-C
vitamins	ascorbic acid	Ascorbique Acide
vitamins	calcitriol	Rocaltrol
vitamins	calcitriol	Calcijex
vitamins	cyanocobalamin	Vitamin B12
vitamins	cyanocobalamin SR	Vitamin B12
vitamins	folic acid	Folic Acid
vitamins	folic acid	Novo-Folacid
vitamins	folic acid	Folique Acide
vitamins	folic acid study	folic acid study
vitamins	hydroxycobalamin	Vitamin B12
vitamins	niacin	Niacin
vitamins	phytonadione	Phytonadione
vitamins	pyridoxine	Vitamin B6
vitamins	pyridoxine	Pyridoxine HCl
vitamins	thiamine	Vitamin B1
vitamins	thiamine	Thiamine Hydrochloride
vitamins	vitamin A	Vitamin A
vitamins	vitamin D2	Drisdol
vitamins	vitamin D3	Vitamin D
vitamins	vitamin D3	D Vi Sol Infant Drops
vitamins	vitamin E	Vitamin E
vitamins	vitamin E	Aquasol E

Copyright © CACR

Copyright © Canadian Association of Cardiac Rehabilitation 2012
To be used with permission only. Not for re-distribution

vitamins	vitamin E	Novo-E (D-Natural)

Copyright © CACR
